# Transient thyrotoxicosis as an initial presentation of rheumatoid arthritis: a case report

**DOI:** 10.4076/1757-1626-2-7310

**Published:** 2009-07-16

**Authors:** Fadi Makdsi, Michael Brit

**Affiliations:** Department of Medicine, University of Tennessee, College of Medicine975 East Third Street, Suite 94, Chattanooga, TN 37403USA

## Abstract

**Introduction:**

Few reports in the literature describe the association of rheumatoid arthritis and transient thyrotoxicosis. We report a case of rheumatoid arthritis and painless thyroiditis presenting simultaneously and acutely with a cluster of symptoms that initially made the diagnosis of rheumatoid arthritis very challenging.

**Case presentation:**

A 41-year-old Caucasian male presented with complaints of malaise and decreased range of motion in the left elbow. Physical examination and laboratory evaluation established the diagnosis of rheumatoid arthritis and painless thyroiditis.

**Conclusions:**

When patients with rheumatoid arthritis present with numerous “extra articular” and constitutional symptoms, evaluation of thyroid disorder with thyroid function test should be considered to help establish the correct diagnosis.

## Introduction

It is well recognized that individuals with one autoimmune disease are more likely to have another autoimmune disorder. Hypothyroidism is the most common thyroid dysfunction that has been reported in association with rheumatoid arthritis (RA). To our knowledge only two reports in the literature describe the association of rheumatoid arthritis and transient thyrotoxicosis [1,2]. We report a case of rheumatoid arthritis and painless thyroiditis presenting simultaneously and acutely with a cluster of symptoms that initially made the diagnosis of rheumatoid arthritis very challenging.

## Case presentation

**Figure 1.**
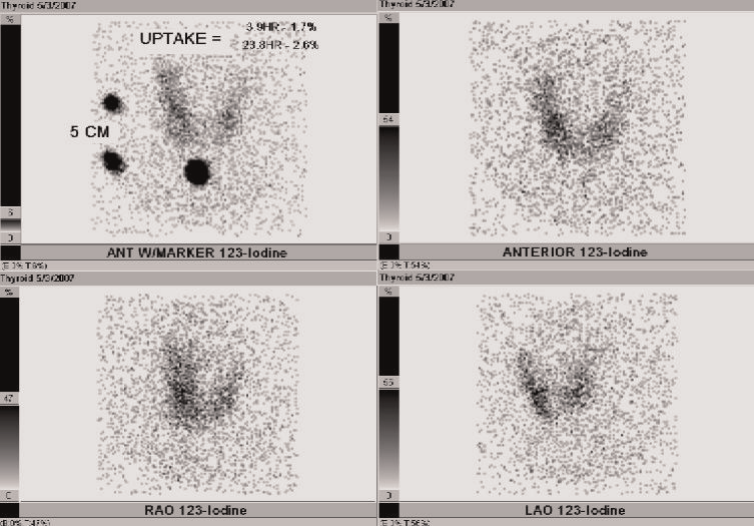
Patient’s thyroid uptake scan (RAIU) shows a reduced twenty four hour uptake and little uptake throughout the thyroid gland.

A 41-year-old Caucasian male presented with complaints of malaise, fatigue and 20 pound weight loss within one month. He also complained of anxiety, palpitation and tremor. He had a two month history of moderate pain and decreased range of motion in the left elbow. He was taking naproxen with no relief. He had no significant past medical history. Physical examination revealed an ill appearing male who was nonfebrile with a heart rate of 110 beats per minute. The thyroid gland was soft, non tender, not enlarged and without nodules. Heart auscultation revealed irregular rhythm but no murmurs. Left elbow was enlarged, warm and slightly tender with limited extension and flexion, but no clear evidence of effusion. The deep tendon reflexes of his extremities were hyperactive. Laboratory evaluation was significant for low TSH: <0.01 mcIU/ml (0.45-6.2), and high free T4: 3.27 ng/dl (0.54-1.24). The titers of thyroglobulin and thyroid peroxidase antibodies were increased: 999 IU/ml (<20), 41 IU/ml (<35) respectively. ESR was normal: 12 mm/hr (0-15). Rheumatoid factor and anti-CCP were both elevated: 105 IU/ml (0-15), 141 units (<20) respectively. EKG demonstrated atrial fibrillation with rapid ventricular response. Left elbow x-ray showed degenerative spurring. Thyroid uptake scan (RAIU) showed a reduced twenty four hour uptake of 2.6% and little uptake throughout the thyroid gland (Figure 1). Treatment with metoprolol was initiated. On the second visit three weeks later his heart rhythm was regular. He complained of morning stiffness and bilateral ankle pain. He had lost five additional pounds. His ankles were swollen, warm and tender with limited extension. He was started on methotrexate (MTX) and prednisone for newly diagnosed RA. Five months from initial presentation, laboratory evaluation revealed normal TSH: 1.79 and free T4:0.88. He remains in euthyroid state on subsequent follow-up.

## Discussion

Rheumatoid arthritis (RA) is a chronic autoimmune disease that primarily affects the joints. Other organs such as the skin, lungs and heart can also be involved. Association of RA with other autoimmune diseases such as an autoimmune thyroid disease has been described [3]. The prevalence of abnormal thyroid function among patients with RA ranges from 7.4% to 34% [4-8]. Hypothyroidism is the most reported thyroid dysfunction in patients with RA [5,6,9]. Hyperthyroidism in patients with RA has been found to be less common than hypothyroidism. Chan et al *(2001)*, found only one patient with subclinical hyperthyroidism among 64 patients with RA (1.6%). The association between RA and hyperthyroid disorders has included transient thyrotoxicosis. Transient thyrotoxicosis can develop with painless thyroiditis when inflammation of the thyroid gland results in hormone leakage. Silent thyroiditis and sporadic thyroiditis have also been used to describe this disorder. Literature has rarely described the concurrence of RA with transient thyrotoxicosis. To the best of our knowledge there are only two reports in the literature that describe the association of rheumatoid arthritis and transient thyrotoxicosis. Murayama et al *(1982)*, reported a patient with history of chronic lymphocytic thyroiditis and RA who develops a transient thyrotoxicosis seven weeks after cessation of steroid therapy. Sakata et al *(1992)*, described a case when a patient was diagnosed simultaneously with RA and silent thyroiditis; the patient’s thyroiditis remained asymptomatic in that case.

Our observation is unique given that painless thyroiditis and RA were presented acutely and simultaneously. Unlike the patient in Sakata’s case, our patient had a significant number of symptoms related to thyroiditis at his presentation; atrial fibrillation, malaise, fatigue, anxiety, palpitation, tremor and 20 pound weight loss. Diagnosis was difficult because clinical signs of transient thyrotoxicosis overshadowed early presentation of RA. Painless thyroiditis was diagnosed based on a normal thyroid exam, a low TSH level, high serum Free T4 and the fact that both levels subsequently normalized. Elevated anti-TPO and anti-TG antibodies, normal ESR, and low radioiodine uptake also helped to confirm the final diagnosis. The acute and concurrent presentation of RA with thyroiditis raised the question of whether this was an instance of two autoimmune diseases being presented simultaneously or did the thyroiditis represent an extra-articular manifestation of RA.

## Conclusions

When patients with rheumatoid arthritis present with numerous “extra articular” and constitutional symptoms, evaluation of a thyroid disorder with thyroid function test should be considered to help establish the correct diagnosis.

## Statement of proprietary interest

The authors hereby state that there are no commercial or proprietary interests of any kind of any drug, device, or equipment mentioned in this study. Neither author has any financial interest of this study.

## Abbreviations

anti-CCP: Anti-cyclic citrullinated peptide antibodies
anti-TG: anti-thyroglobulin antibodies
anti-TPO: anti-thyroid peroxidase antibodies
RA: rheumatoid arthritis
RAIU: radioactive iodine uptake scan
